# Land Suitability Assessment on a Watershed of Loess Plateau Using the Analytic Hierarchy Process

**DOI:** 10.1371/journal.pone.0069498

**Published:** 2013-07-26

**Authors:** Xiaobo Yi, Li Wang

**Affiliations:** 1 College of Resources and Environment, Northwest A&F University, Yangling, Shaanxi, China; 2 State Key Laboratory of Soil Erosion and Dryland Farming on the Loess Plateau, Northwest A&F University, Yangling, Shannxi, China; University of Florida, United States of America

## Abstract

In order to reduce soil erosion and desertification, the Sloping Land Conversion Program has been conducted in China for more than 15 years, and large areas of farmland have been converted to forest and grassland. However, this large-scale vegetation-restoration project has faced some key problems (e.g. soil drying) that have limited the successful development of the current ecological-recovery policy. Therefore, it is necessary to know about the land use, vegetation, and soil, and their inter-relationships in order to identify the suitability of vegetation restoration. This study was conducted at the watershed level in the ecologically vulnerable region of the Loess Plateau, to evaluate the land suitability using the analytic hierarchy process (AHP). The results showed that (1) the area unsuitable for crops accounted for 73.3% of the watershed, and the main factors restricting cropland development were soil physical properties and soil nutrients; (2) the area suitable for grassland was about 86.7% of the watershed, with the remaining 13.3% being unsuitable; (3) an area of 3.95 km^2^, accounting for 66.7% of the watershed, was unsuitable for forest. Overall, the grassland was found to be the most suitable land-use to support the aims of the Sloping Land Conversion Program in the Liudaogou watershed. Under the constraints of soil water shortage and nutrient deficits, crops and forests were considered to be inappropriate land uses in the study area, especially on sloping land. When selecting species for re-vegetation, non-native grass species with high water requirements should be avoided so as to guarantee the sustainable development of grassland and effective ecological functioning. Our study provides local land managers and farmers with valuable information about the inappropriateness of growing trees in the study area along with some information on species selection for planting in the semi-arid area of the Loess Plateau.

## Introduction

Sandy desertification and soil erosion are two of the most serious problems affecting China's water and land resources. The World Bank has suggested that potentially more than 331 million hectares of land are susceptible to desertification (about one third of the area of China) while about 262 million hectares are actually affected [1]. Previous studies have shown that soil erosion affects about 360 million hectares of land in China, which is about 38% of its total area, and this proportion is more than three times the global average [2]–[3]; land in China is considered to be amongst the most severely eroded in the world. For example, on the Loess Plateau, erosion rates are about 8000–25,000 tonnes km^−2^ year^−1^ in the gully areas of Shanxi and Shaanxi Provinces. Intensive cultivation of the steep hillsides has resulted in the loss of an estimated 1.6 billion tonnes of soil annually to the Yellow River [4]. In addition, dry conditions combined with the fine texture of the loess soil make the area very susceptible to dust storms. Due to the severe soil erosion and sandy desertification, the eco-environment of the Loess Plateau has been severely degraded, which has seriously affected sustainable development in the region [5]. With the environmental goals of reducing soil erosion and desertification, the Chinese Central Government launched the Sloping Land Conversion Program (SLCP, also known as Grain for Green or Grain to Green) in the late 1990s, with the intension of increasing the country's forest and grassland cover by retiring steeply sloping and marginal land from agricultural production [6]–[9]. This program aimed to restore degraded ecosystems, reduce poverty and assist rural households to move towards more sustainable economic activities [2], [10].

However, after more than 10 years, this large-scale ecological-recovery project has faced some key problems that have limited the successful development of the current SLCP policy [11]. Surveys and case studies of SLCP have consistently identified insufficient technical support and arid conditions as being the key constraints to achieving program goals [3]. For example, in the north part of the Loess Plateau, the potential for converting cropland into forests has been over estimated: tree species have been planted in areas that are better suited to growing shrubs or grass, and species with high water requirements have been planted in areas where drought-tolerance is required. The result has been soil desiccation and the development of dry soil layers because the planted trees exploited water stored in the deeper soil layers and prevented them from being recharged by rainwater [12], [7]. Consequently, once the stored water was exhausted, the limited precipitation was insufficient to maintain normal growth in the re-vegetated areas [13], leading to the vegetation dying or the production of stunted trees (colloquially referred to as “little old man trees”, which are only about 20% of the normal height for their age) [14]–[17]. Thus, implementing the SLCP policy on the Loess Plateau requires careful consideration of several factors that have a significant effect on land use change and land suitability associated with carrying out the re-vegetation program. Currently, China is facing increased environmental pressures with shortages of water potentially limiting development, especially in its dryer northern and western regions, including the Loess Plateau [12]. In order to reduce soil water depletion and implement a successful re-vegetation program for environmental improvement on the Loess Plateau it is essential to identify appropriate species to plant. It is important to evaluate the land suitability and to develop land use plans that maximize reduction in soil erosion while at the same time minimizing water yield reduction, thus ensuring the sustainable growth and succession of vegetation. Only the proper implementation of the SLCP and successful re-vegetation can make a real contribution to efforts being made to combat the urgent environmental problems of soil erosion and desertification, as well as of climate change and loss of biodiversity, currently confronting the Loess Plateau, in particular, and China as a whole. In this study, the analytic hierarchy process (AHP) was used to assess the suitability of cropland, grassland and forestland in the Liudaogou watershed, and the main limiting factors for different land use options were quantitatively analyzed. The AHP has been widely used b**y** decision-makers and researchers. It is a mathematical method, developed by Saaty in 1977 [18] and improved by the same author in 1980 [19], for analyzing complex decisions involving many criteria [20]. It has been widely used in site selection, suitability analysis, regional planning, and land consolidation analysis [21]–[22].The success of the AHP as a practical and reliable method is highlighted by its extensive application in the past two decades [23]–[24]. Furthermore, its simplicity in relation to its power was a significant factor in the choice for its use in the presented study, and all the mentioned factors ensured that the study objectives would be successfully achieved. The objectives of the study are to determine which land use is best suited to the re-vegetation program by assessing the land suitability, to identify the constraints for future land conservation, and to provide a scientific basis for decision-making in the successful implementation of the Sloping Land Conversion Program, not only for the study's watershed but across the whole Loess Plateau.

## Materials and Methods

### Study area

**Figure 1:**
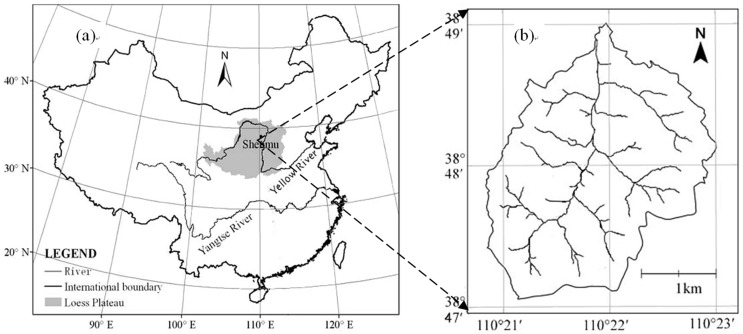
Location of the study site. (a) China; (b) the Liudaogou watershed.

This study was conducted in the Liudaogou watershed, located in Shenmu County, Shaanxi Province, China (110°21′–110°23′E, 38°46′–38°51′N; Fig. 1). The Liudaogou watershed covers 6.9 km^2^ and is located at the center of the wind-water erosion crisscross region in the north part of the Loess Plateau. This area suffers its most serious water erosion in summer and autumn and its most serious wind erosion in winter and spring [25]–[26]. The watershed is representative of the ecotone between the grass–pastoral and the agricultural areas, in the transitional zone between the desert aeolian deflation zone (the Mu Us Desert) and the loess hilly area (Loess Plateau), as well as being between the arid and the semi-arid regions. For these reasons, the Shenmu Erosion and Environmental Research Station (SEERS) of the Chinese Academy of Sciences was built within the watershed [27]. The study area is located at altitudes ranging from 1080 to 1270 m above mean sea level and has a semiarid continental monsoon climate, with a mean annual temperature of 8.4°C. The monthly mean temperature ranges from 9.7°C in January to 23.7°C in July. The mean annual precipitation is 437 mm, 77% of which occurs from June to September. Mean annual potential evapotranspiration can be as high as 1800 mm, which would result in a water deficit of 1350 mm year^−1^. The area has a deep (up to 100 m) loess layer, which originated during the Quaternary period. The dominant soil type (cultivated loessial soil), is a Ust-Sandic Entisol, which is loess-derived and is consequently easily eroded by both water and wind.

### Analytical hierarchy process (AHP)

**Figure 2:**
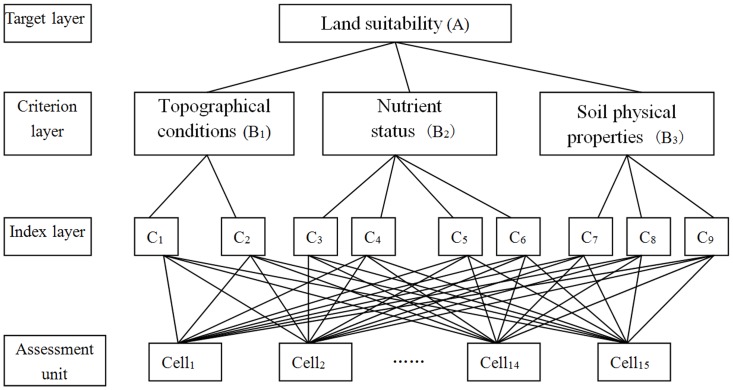
Hierarchical structure of land suitability.

The application of AHP for making a decision about land suitability in this study involved four main steps, as follows:

The first step was to decompose the decision problem into a hierarchical structure where the attributes and plans were present as inter-related elements. Based on a qualitative analysis of the environment in the study area, the final hierarchical structures were separated into four levels (Fig. 2). The first level was the overall land suitability. The second level was composed of subsystems: geological and topographical conditions; nutrient status; and soil physical properties. The third level consisted of the specific factors that affected the land suitability. The fourth level comprised each assessment unit (cell).

Elevation (C_1_) and slope gradient (C_2_) were selected to represent geological and topographical conditions (B_1_). Slope is a crucial factor affecting vegetation structure and soil erosion on the Loess Plateau. Variation in elevation has an impact on soils, microclimatic effects, and other processes that could affect land suitability. Soil organic matter (C_3_), total nitrogen (C_4_), available phosphorous (C_5_), and available potassium (C_6_) were selected as factors that represented nutrient status (B_2_). These four nutrition factors are closely related to land use and cover change, and understanding the effects of land use change on soil organic matter and nitrogen is important to sustainable management of land resources and associated watershed processes, as well as regional responses to global climatic change [28]. Three factors were selected to represent the soil physical properties. Soil bulk density (C_7_), soil texture (C_8_) and soil water content (C_9_) strongly influence plant growth and land use, and soil properties and plant recovery processes have distinct characteristics in the typical desertified sandy land of the Loess Plateau [17]. Socio-economic factors were not considered in this study because it was limited to a small watershed where such factors are generally less important than the physical ones.

The second step involved a pair-wise comparison of the elements based on a nine point weighting scale; this generated the input data (Table 1). The comparison was carried out for each decision element at 1–(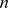
–1) levels, where 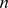 was the matrix size. A matrix was generated as a result of the pair-wise comparisons and weights for the criteria were derived from these calculations.

**Table 1:** AHP pair-wise comparison scale for variables *i and j*.

Intensity of the relative importance	Definition
1	Equal importance of *i* and *j*
3	Moderate importance of *i* over *j*
5	Strong importance of *i* over *j*
7	Very strong importance of *i* over *j*
9	Extremely strong importance of *i* over *j*
2, 4, 6, 8	Intermediate values

A matrix of scores could be developed from the comparisons, given by
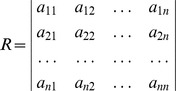
where 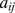 indicated how much more important the 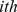 objective was than the 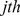 objective, while making a suitable material handling/equipment selection decision. For all 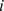 and 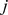, it was necessary that 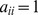 and 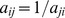. The possible assessment values of 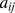 in the pair-wise comparison matrix, along with their corresponding interpretations, are shown in Table 1.

The scores were normalized by dividing each element within the matrix by the sum of the column 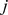, to create a normalized matrix, 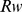:
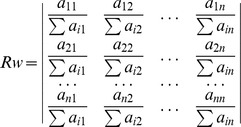



The weight associated with each objective (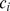) could be estimated as the mean of the normalized scores in row 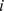 of the 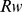 matrix. Hence, 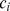 was calculated to give a matrix of weights, *C*:
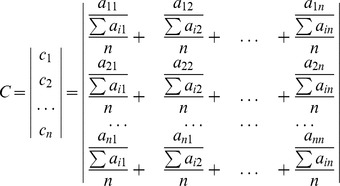
where 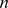 is the number of objectives being compared, and the 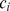 value indicates the relative degree of importance (weight) of the 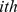 objective.

The third step was to check for the consistency of the weight values (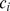) underlying the theoretical validity of the comparison matrix. In order to determine consistency, the consistency vector (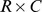 matrix) and 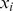 were calculated as:
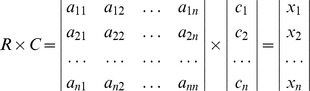
The eigenvalue of the pair-wise comparison matrix, 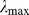, was then estimated using the following equation:
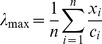
An approximation to the consistency index (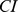) was calculated and the consistency judgment was checked for the appropriate value of 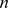 by 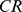. 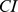 and 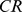 were calculated as [18]:
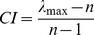


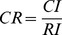
where 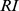 is the random consistency index. The 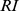 values for different numbers of 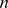 are listed in Table 2.

**Table 2:** Random consistency index for AHP.

*n*	1	2	3	4	5	6	7	8	9
*RI*	0.00	0.00	0.58	0.90	1.12	1.24	1.32	1.41	1.45

The rule commonly used in the AHP was applied, whereby if 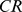 was less than 0.10 (i.e. 10%), the degree of consistency was acceptable; and if it was greater than 0.10 it was considered that there were significant inconsistencies and, in such a case, the AHP would not produce meaningful results [29], and it would be necessary to review and improve the judgments.

The fourth step was a complete evaluation based on the rating of the final weights in the decision plan. Matrixes for layers B and C and for C and D were generated using the same method as that used for layers A and B. By comparing the final values by simple rankings, the weights of all elements in each level of the hierarchy relative to the entire level could be obtained. These in turn were all ranked, and were carried from the upper layer to the lower layer. The combined weight (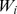) of each assessment factor was determined for the integrated assessment of land suitability in the Liudaogou watershed.

### Membership value standardization for assessment factors

In the process of land suitability assessment, a primary step was to ensure a standardized measurement system for all the factors considered. Since those factors have different standards of measurements, they had to be standardized to a uniform rating scale; in this study the scale was between 1 and 4 for ease of analysis. Assigning values to specific factors required specific decision rules in the form of thresholds for each factor. Various statistical and empirical guidelines from the related national codes and literature were used to determine the boundary values. As a general guideline, a positive correlation between the value awarded and suitability was employed. The class boundaries and standardized measurements employed for each factor are given for arable land in Section 3.1.1 and for forestland and grassland in Section 3.2.1. The integer numbers ranging from 1 to 4 were assigned to high, moderate, marginal and unsuitable classes, respectively. The next step involved assigning a new value for the degree of membership of every attribute to each index at each level. All these new values ranged from 0 to 1 where 0 indicated a poor fit and 1 indicated a perfect fit for membership of an attribute to a given index.. The membership degree function (*F_i_*) of each assessment factor is given as follows:

The membership degree of soil organic matter, total nitrogen, available phosphorus and available potassium to the soil nutrient status criterion were calculated using the following equations:
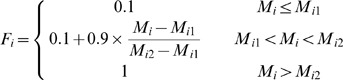
where 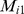 and 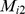 are the lower and upper limits of the value range of a factor.

The membership degree of slope gradient was given by:
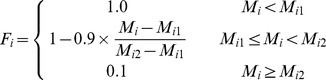



The membership degree of physical clay content was given by:
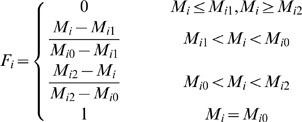
where 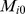 means the best physical clay content in the study area.

The membership degree of altitude was given by:
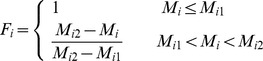



The degree of membership of soil water content and bulk density was given by:
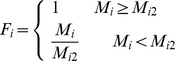



### Assessment of land suitability

The integrated classification of land suitability for the study area was obtained by integrating the combined weight (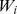) and the total degree of membership for different factors (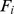) as follows:
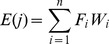



The maximum membership principle was adopted to define the comprehensive classification of land suitability. If 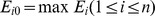, the comprehensive classification was 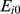.

### Data

Field survey and soil sampling were conducted during August and September 2011. The land use status in 1993 and 2011 is presented in Table 3. When compared with 1993, there was significantly less arable land in 2011, whilst there had been increases in the extent of grassland, land that had been build on and land that was not in use. The main reason was the construction of four collieries during this period; this resulted in the transfer of the work force (local farmers) from traditional cultivation to the coal industry [30]. Based on land use and topography, we selected 15 assessment units: three for representing arable land, five for forestland and seven for grassland. Details of the assessment units are presented in Table 4.

**Table 3:** Land use in the Liudaogou watershed (%) in 1993 and 2011.

Year	Arable land	Forestland	Grassland	Others2)
19931)	31.3	25.7	39.6	3.4
2011	16	26	44	14

Note:

^1)^ Data cited are from Yang et al. (1994).

^2)^ Refers to built-up and unused land.

**Table 4:** Detailed information about the assessment units.

Assessment unit	Land use	Slope gradient (°)	Altitude (m)	Soil organic matter (g/kg)	Total nitrogen (g/kg)	Available phosphorus (mg/kg)	Available potassium (mg/kg)	Bulk density (g/cm^3^)	Soil texture (physical clay content ≤0.01 mm) (%)	Soil water content (%)	Main species
1	Arable land	7	1170	14.29	0.41	4.64	203.78	1.60	17.52	9.45	*Solanum tuberosum*
2	Arable land	5	1127	22.26	0.39	15.83	420.90	1.57	12.99	12.16	*Setaria italica*
3	Arable land	0	1155	16.02	0.24	7.36	244.80	1.59	11.37	12.09	*Zea mays*
4	Forestland	15	1195	9.54	0.22	5.12	269.09	1.53	6.01	3.71	*Populus simonii*
5	Forestland	14	1201	13.84	0.35	5.87	279.82	1.60	7.07	4.46	*Populus simonii*
6	Forestland	10	1170	12.50	0.26	5.17	176.56	1.54	5.14	2.00	*Pinus tabulaeformis*
7	Forestland	12	1154	5.80	0.07	3.77	72.55	1.55	6.59	3.78	*Populus simonii*
8	Forestland	17	1175	3.08	0.05	3.14	133.91	1.56	6.86	3.91	*Populus simonii*
9	Grassland	17	1160	2.90	0.12	3.06	119.29	1.60	9.21	3.90	*Stipa bungeana*
10	Grassland	15	1196	1.99	0.08	2.46	92.97	1.58	19.13	3.51	*Medicago sativa*
11	Grassland	20	1189	8.14	0.16	4.58	116.34	1.58	2.27	3.64	*Medicago sativa*
12	Grassland	30	1204	6.23	0.13	3.95	297.40	1.61	12.45	8.60	*Lespedeza daurica*
13	Grassland	10	1237	11.02	0.22	7.28	157.24	1.59	39.50	9.54	*Stipa bungeana*
14	Grassland	9	1152	6.38	0.14	4.11	85.04	1.59	18.86	8.17	*Stipa bungeana*
15	Grassland	18	1197	2.46	0.08	2.98	186.94	1.57	20.52	8.50	*Stipa bungeana*

Altitude was measured in the field by double frequency RTK-GPS. Slope gradient was determined using a compass incorporating an inclinometer. Sampling transects were placed along the slope in each assessment unit. For each transect, 3 plots (15×15 m) were established for the field investigation and sampling. In each sampling plot, a 1.0 m long×0.7 m wide×0.5 m deep pit was dug to allow measurements of soil bulk density. Samples were collected using a 5.0 cm diameter by 5.0 cm long stainless cutting ring with which samples were collected from the top 20 cm of the soil profile. Soil samples in the ring were extracted and the roots were carefully removed by hand. The soils were dried at 105°C to constant weight and bulk density was calculated by dividing the dry mass by the known volume. Soils were also collected with a soil auger (4 cm-diameter) to estimate profile water content in 10 cm layers to a depth of 40 cm. Soil water content was determined by oven-drying samples at 105°C.

Composite samples of about 1 kg from each assessment unit were collected and then air-dried and ground to pass through 1.00 mm and 0.25 mm nylon screens prior to laboratory analysis. The sieved soil was used to measure soil particle composition (physical clay content) and soil organic matter (SOM) , respectively. Particle composition was measured by the laser diffraction technique using a MasterSizer 2000 (Malvern Instruments, Malvern, England), equipped with a low-power (2 mW) Helium-Neon laser with a wavelength of 633 nm as the light source. Soil organic carbon (SOC) was measured by an Elementar Vario EL element analyzer and soil organic matter (SOM) was obtained by multiplying SOC content values by 1.723.

Soil total nitrogen (TN) was measured following the Kjeldahl digestion method [31]. Total phosphorus (TP) was determined colorimetrically after wet digestion with sulfuric acid and perchloric acid, and available phosphorus (AP) was extracted following the Olsen bicarbonate extractable P method [32]. Available K was determined by the ammonium acetate extraction method [33].

## Results and Discussion

### Suitability assessment for arable land

#### Standardized evaluation factors for arable land

Arable land suitability was divided into 4 classes, i.e. highly suitable (S1), moderately suitable (S2), marginally suitable (S3) and unsuitable (N). All factors were standardized to a uniform rating scale for ease of analysis. Standardized values for specific factors required specific decision rules in the form of thresholds for each factor. Various statistical and empirical guidelines from the related national–provincial codes and literature were used to determine the boundary values. In addition, twelve experts were invited to act as the decision makers to guarantee the reliability of boundary values. The experts involved were ecologists, pedologists, geographers, forest and grass managers, land resource experts and environmental protection experts; all have undertaken related studies of vegetation restoration and ecological– environmental issues in the study area and all are familiar with the Loess Plateau. Thus, they were able to suggest reasonable values for the thresholds. As a general guideline, there was a positive correlation between the value assigned and suitability. The class boundaries and standardized measurements applied for each factor are shown in Table 5.

**Table 5:** Land characteristics, thresholds and degree of suitability for arable land.

Land characteristics	Suitability			
	High (S1)	Moderate (S2)	Marginal (S3)	Unsuitable (N)
Slope gradient (°)	≤5	5∼15	15∼25	>25
Altitude (m)	≤1170	1170∼1195	1195∼1220	1220∼1273.9
Soil organic matter (g/kg)	≥20	13∼20	6∼13	<6
Total nitrogen (g/kg)	≥0.28	0.16∼0.28	0.05∼0.16	<0.05
Available phosphorus (mg/kg)	≥15	9∼15	3∼9	<3
Available potassium (mg/kg)	≥250	150∼250	50∼150	<50
Bulk density (g/cm^3^)	-	-	-	>1.60
Soil texture (physical clay content) (%)	15	-	-	>30 or <10
Soil water content (%)	≥12	10∼12	8∼10	<8

#### Distribution of suitable cropland and associated statistics

Based on the method described above, suitable cropland classes were calculated and the main limiting factors were identified for each assessment unit (Table 6). Assessment units 1 and 3 in arable land and unit 14 in grassland were considered to be of marginal suitability, assessment unit 2 in arable land was classified as being of moderate suitability, and the other units from forest and grasslands were classified as being unsuitable for crops. Generally, an area of 0.40 km^2^, accounting for 6.7% of the total area of the watershed (not including the built-up and unused lands), was classified as being moderately suitable for crops, and 1.19 km^2^ (20.0%) as marginally suitable for crops (Table 7). In total, the area suitable for crops comprised about 26.7% of the watershed, so the area unsuitable for crops accounted for 73.3% of the watershed. The main factors restricting cropland development were soil physical properties and soil nutrients. First, the soil organic matter content and total nitrogen were generally low. This was particularly true in forest and grasslands, where the total nitrogen ranged between 0.05 and 0.35 g/kg (on average 0.17±0.09 g/kg), and was significantly lower than the generally reported values for soils (0.48–0.91 g/kg; on average 0.71) in the center of the Loess Plateau [34]. Soil organic matter was in the range 1.99–13.84 g/kg (on average 6.99±4.05 g/kg), and lower than the generally reported values for soils (7.0–15.0 g/kg; on average 11.0) in the center of the Loess Plateau [35]. Secondly, soil texture and soil water content obviously affected the land's suitability to grow crops in the watershed. In forestland in particular, the clay content was significantly lower than that of crop and grasslands, suggesting that planting trees adversely affected the soil structure in the study area. Our data were consistent with those presented in previous studies [26], [30] indicating that species such as *Populus simonii* can cause soil degradation. The watershed is located in the ecotone between arid and the semi-arid regions, and soil water is always a limiting factor for plant growth because of the low levels of precipitation (about 437 mm) and high potential evapotranspiration (1800 mm). In forestland, soil water content ranged between 2.00% and 4.46%, which was close to the wilting point. Grasslands also exhibited soil water deficits compared with the croplands, although soil water content was higher than that in forestlands. Previous studies have shown that planting *P. simonii* (a tree species) and *Medicago sativa* (alfalfa, a forage legume) could cause large reductions in soil water content due to their high water consumption, and this could result in the formation of dry soil layers on land where they have been planted [36]–[37]. Usually, soil drying due to high plant water use adversely affects soil physical properties, for example, reducing aggregate stability and soil surface roughness [38]–[39]. Thus, soil water deficit and soil physical properties may have a compounding effect.

**Table 6:** Cropland suitability and limiting factors for each assessment unit.

Assessment unit	Combined weight	Grade	Limiting factors
1	0.709	S3	-
2	0.989	S2	-
3	0.795	S3	-
4	0.466	N	Soil water and texture
5	0.619	N	Soil water and texture
6	0.501	N	Soil water and texture
7	0.259	N	Soil water, texture and organic matter
8	0.244	N	Soil water, texture and organic matter
9	0.287	N	Soil water, texture and organic matter
10	0.260	N	Soil water, organic matter
11	0.347	N	Soil water and texture
12	0.390	N	Bulk density
13	0.607	N	Soil texture and slope gradient
14	0.427	S2	-
15	0.371	N	Soil organic matter and available P

**Table 7:** Areas of land suitable for growing crops in the Liudaogou watershed.

Grade	Area (km^2^)	Percentage (%)
S1	0	0
S2	0.40	6.7
S3	1.19	20.0
N	4.34	73.3

It is noteworthy that available phosphorus was in the range 2.46–5.87 mg/kg (on average 4.29±1.39 mg/kg) in forest and grass lands, and was, therefore, very much higher than generally reported for soils (0.91–1.76 g/kg; on average 1.10) in the center of the Loess Plateau. Available potassium was in the range 85.04–297.40 mg/kg (on average 165.60±78.49 mg/kg), which is also significantly higher than generally reported for soils (77.2–170.0 mg/kg) in the center of the Loess Plateau [35]. Therefore, these two nutrients were not the limiting factors for cropland development in the watershed.

### Suitability assessment for forest and grassland

#### Standardized evaluation factors for forest and grassland

As for the arable land suitability assessment, forest and grassland suitability was also divided into 4 categories: highly suitable (S1), moderately suitable (S2), marginally suitable (S3) and unsuitable (N). The class boundaries and standardized measurements applied for each factor are shown in Table 8. Altitude is not a limiting factor for forest and grassland development.

**Table 8:** Land characteristics, thresholds and degree of suitability for forest and grasslands.

Land characteristics	Forest or grassland	Suitability			
		High (S1)	Moderate (S2)	Marginal (S3)	Unsuitable (N)
Slope gradient (°)	Both	≤15	15∼25	25∼35	>35
Soil organic matter (g/kg)	Forest	≥7.97	5.5∼7.97	3.08∼5.5	<3.08
	Grass	≥5.41	3.50∼5.41	1.99∼3.5	<1.99
Total nitrogen (g/kg)	Forest	≥0.19	0.12∼0.19	0.05∼0.12	<0.05
	Grass	≥0.11	0.08∼0.11	0.05∼0.08	<0.05
Available phosphorus (mg/kg)	Forest	≥4.62	3.88∼4.62	3.14∼3.88	<3.14
	Grass	≥3.66	2.52∼3.66	2.46∼2.52	<2.46
Available potassium (mg/kg)	Forest	≥250	150∼250	50∼150	<50
	Grass	≥250	150∼250	50∼150	<50
Bulk density (g/cm^3^)	Both	-	-	-	>1.65
Soil texture (physical clay content) (%)	Both	15	-	-	>40 or <5
Soil water content (%)	Forest	≥15	11.5∼15	8∼11.5	<8
	Grass	≥4.78	4.14∼4.78	3.50∼4.14	<3.50

#### Distribution of forest and grassland suitability

Only assessment unit 6 in the forestland and unit 11 in the grassland were classified as being unsuitable for grassland (Table 9). The limiting factor for unit 6 was the low soil water content resulting from the high water consumption by tree species, and the limiting factor for unit 11 was soil texture because of its very low clay content (Table 4). Overall, an area of 0.79 km^2^, accounting for 13.3% of the total area of the watershed, was classified as highly suitable for grassland, 1.18 km^2^ (20.0%) was classified as being moderately suitable for grassland, and 3.16 km^2^ (53.4%) as being marginally suitable for grassland (Table 10). In total, the area suitable for grassland was about 86.7% of the watershed, with the remaining 13.3% being unsuitable. This result is consistent with a previous study suggesting that development of vegetation in the watershed should focus on grassland [30]. Hou et al. [27] reported that the watershed is located within the boundary of the 400 mm rainfall isoline, representing the demarcation between cropping and pastoral regions. From the perspective of the climate, the watershed is more suitable for the growth and development of herbaceous plants than for trees. It is also notable that the area is not entirely appropriate for grassland, with 53.4% being classified as only marginally suitable. Besides soil water content and soil texture, total nitrogen may be a subsidiary factor limiting the suitability of this land for use as grassland – it is present at low levels in forest and grasslands (Table 11).

**Table 9:** Grassland suitability and limiting factors for each assessment unit.

Assessment unit	Combined weight	Grade	Limiting factors
1	0.984	S2	-
2	0.995	S1	-
3	0.989	S2	-
4	0.926	S3	-
5	0.965	S1	-
6	0.821	N	Soil water
7	0.751	S3	-
8	0.538	S3	-
9	0.683	S3	-
10	0.441	S3	-
11	0.865	N	Soil texture
12	0.945	S3	-
13	0.948	S2	-
14	0.948	S3	-
15	0.632	S3	-

**Table 10:** Areas suitable for use as grassland in the Liudaogou watershed.

Grade	Area (km^2^)	Percentage (%)
S1	0.79	13.3
S2	1.18	20.0
S3	3.16	53.4
N	0.79	13.3

**Table 11:** Forestland suitability and limiting factors for each assessment unit.

Assessment unit	Combined weight	Grade	Limiting factors
1	0.900	S3	-
2	0.952	S2	-
3	0.945	S2	-
4	0.806	N	-
5	0.820	N	-
6	0.756	N	Soil water
7	0.423	N	Organic matter and soil water
8	0.249	N	Organic matter and soil water
9	0.507	N	Organic matter, soil water and total nitrogen
10	0.458	N	Organic matter, soil water and total nitrogen
11	0.708	N	Soil water
12	0.603	N	Organic matter and soil texture
13	0.865	S3	-
14	0.658	S3	-
15	0.556	N	Organic matter and total nitrogen

With respect to forestland suitability, assessment units 2 and 3 in the arable land were classified as being moderately suitable, assessment unit 1 in the arable land and units 13 and 14 in the grassland were considered marginally suitable, and other units in the forest and grasslands were unsuitable for use as forest. Overall, an area of 0.79 km^2^, accounting for 13.3% of the total area of the watershed, was classified as moderately suitable for forestland and 1.19 km^2^ (20.0%) as marginally suitable for forestland; the remaining 3.95 km^2^, accounting for 66.7% of the watershed was unsuitable for forest (Table 12).

Like cropland, the main factors restricting the establishment of forest were soil water content, organic matter and total nitrogen. This means soil water and nutrition conditions were not suitable for widespread afforestation. The slope gradients of the arable and grassland assessment units that were suitable for forest were all below 10u, indicating that slope gradient may be a subsidiary limiting factor with respect to forests. Usually, steep slopes are associated with low soil water content, with increasing soil erosion and decreasing infiltration [40]–[42].

**Table 12:** Areas of land suitable for forest growth in the Liudaogou watershed.

Grade	Area (km^2^)	%Percentage ()
S1	0	0
S2	0.79	13.3
S3	1.18	20.0
N	3.95	66.7

### Implications for the Sloping Land Conversion Program

On the Loess Plateau, rapid population growth and an economic boom, coupled with severe soil erosion and desertification, have led to deterioration of the natural environment and a reduction in biodiversity [43], [4]. The Sloping Land Conversion Program is, therefore, considered to be a necessary step for increasing vegetation cover and restoring ecosystem service functions, thus promoting sustainable environmental and economic development. However, during implementation of the SLCP, tree planting was overemphasized, resulting in inappropriate planting schemes [34]ü. L et al. [8]; reported that the large areas converted from farmland to woodland have resulted in decreased regional water yield as the climate warms and dries on the Loess Plateau. Successful ecological rehabilitation programs have, thus, been largely dependent on innovative ecosystem management systems and technical support. Land suitability evaluation indicates that the Liudaogou watershed is suited to grass growth and is unsuitable for forest growth on the sloping land this is due mainly to water and nitrogen deficits. In fact, there is still 1.79 km**^2^**% (26 of the total area) of forestland in the watershed, with *Populus simonii* and *Pinus tabulaeformis* that were mainly planted in the late 1970s and after 1999 [27], [30]. Hou [27] and Yang et al. [44] reported that **t**he forest in the Liudaogou watershed could not function as a stable forest ecosystem. The height of the 20-year-old *Populus simonii*–– trees averaged about 46 m (the smallest just 2 m), the diameter at breast height (DBH) was about 56 cm, and the volume of timber was just 0.0031 m^3^ per individual. In general, the growth of *Populus simonii* stopped after about 15 years. Under normal circumstances, the height of a 20-year-old *Populus simonii*≥ tree would be more than 20 m with DBH 15 cm. Due to limited cover and poor growth, the *Populus simonii*“” forest is very restricted in its ecological function. It was originally planted as a part of a shelterbelt in the Three North Protective Forest Program, aiming to combat desertification and soil erosion in the Northwest of China [45]–[46]. However, according to a survey conducted by Hou et al. [47], the wind erosion depth in the *Populus simonii*–× forestland amounts to 1.914.68 cm/a, corresponding to 1.910^4^–×4.710^4^ m^3^;– soil loss per year. Compared with the natural grass vegetation, it does not prevent wind erosion, but greatly accelerates it. The main reasons for the stunted tree growth are: (1) there is insufficient soil water available to maintain normal growth rates (2) there is insufficient soil fertility (e.g. besides low total nitrogen, the Soil Organic Carbon Density is about 1.182.81 kg/m^2^–, significantly lower than the national mean values for soils of 11.5212.04 kg/m^2^) [48];– and (3) there is insufficient management because of the very limited economic value of forest local farmers are more willing to devote themselves to coal-mining which generates a much higher income.

%Although our forestland suitability assessment indicated that about 33.3 of total area is suitable for forest growth (Table 12), such areas are currently arable land and some grassland with gentle gradients (Table 4). Compared to tree species, crop and grass species have shallow roots and consume relatively little water [30];, so do not deplete soil water to any great depth however, the current arable lands are dammed, terraced fields that collect valuable rainfall because of the low soil erosion rates. Therefore, those arable lands and grasslands with gentle gradients were classified as being moderately or marginally suitable for forest development. We predict that planting trees in these areas would cause soil water depletion because of high water consumption combined with the low precipitation rates in the watershed.

%The grassland suitability assessment indicated that about 86.7 of the area of the watershed is suitable for grass growth, thus the SLCP in the watershed should focus on conversion of cropland to grassland. Wang et al. [17]>– reported that soil physical properties such as bulk density, hydraulic conductivity, mean weight diameter, and the stability of 1 mm macro-aggregates have been significantly ameliorated in the 020 cm soil layer under secondary natural grasslands, implying that natural grass (*Stipa bungeana*– Trin.) restoration is an appropriate and sensible approach to re-vegetation in the windwater erosion region of the northern Loess Plateau of China. Planting alfalfa (*Medicago sativa*) and korshinsk peashrub (*Caragana korshinskii*) has no effect on the soil physical conditions, and may even reduce bulk density and soil permeability because of their very high water consumption with deep and vigorous root systems. Therefore, care is required when selecting species for re-vegetation to promote shifts from arable land to grasslands in a more environmentally compatible manner. Non-native grassland species, such as the korshinsk peashrub and alfalfa, have high water requirements and should be avoided when choosing species to plant in the watershed.

## Conclusion

Land use management involves complex decision-making that requires an understanding of many factors. This paper presents the AHP as a decision support tool for use when selecting an appropriate land use, which is an important issue for the sustainable development of the Liudaogou watershed. Based on the AHP, grassland was found to be the most suitable land-use type to support the aims of the Sloping Land Conversion Program in the watershed. Under the constraints of soil water shortage and nutrient deficit, crops and forests were considered to be inappropriate land uses in the study area, especially on sloping land. All forest should be converted to grassland because continued growth of trees will damage the soil water environment and increase desertification problems. When selecting species for re-vegetation, non-native grass species with high water requirements should be avoided so as to guarantee the sustainable development of grassland and effective ecological functioning. In the future, the area of abandoned cropland is likely to increase rapidly due to the government policy embodied in the Sloping Land Conversion Program. Our study provides local land managers and farmers with valuable information about the inappropriateness of growing trees in the study area along with some information on species selection for planting in the semi-arid area of the Loess Plateau.
