# Correction: Land Suitability Assessment on a Watershed of Loess Plateau Using the Analytic Hierarchy Process

**DOI:** 10.1371/annotation/24cc27d5-2941-40d3-8d57-adaa9cccde61

**Published:** 2013-12-30

**Authors:** Xiaobo Yi, Li Wang

This paper was republished on December 23rd 2013 due to missing content. Please see the completed paper on the webpage. 

